# Fundus changes after cardiac surgery

**Published:** 2020

**Authors:** Miriam Rahhal-Ortuño, Alex Samir Fernández-Santodomingo, Marina Aguilar-González, Emma Marín-Payá, Rafael Martínez-Costa

**Affiliations:** * Department of Ophthalmology, Hospital Universitari i Politecnic La Fe, Valencia, Spain

**Keywords:** cardiac surgery, posterior cerebral artery occlusion, retinal ischaemia

## Abstract

A Caucasian male with known severe aortic stenosis was referred to our Ophthalmology Department after undergoing cardiac surgery using extracorporeal circulation. Signs of retinal ischaemia were found during fundus examination and neuroimaging showed posterior cerebral artery occlusion.

## Introduction

Extracorporeal circulation involves a variety of changes in the organism’s hemodynamics involving vasodilation and vasoconstriction. Retinal tissue is an easy target for complications to occur as its circulation is an end-arterial system.

## Case presentation

A 53-year-old Caucasian male was referred to our department after undergoing cardiac surgery as he reported that he was not able to see his left visual field. He suffered from severe aortic stenosis associated with mitral insufficiency and dilation of the ascending aorta. Cardiac surgery was performed using extracorporeal circulation and lasted for over 450 minutes. It involved a Bentall procedure and mechanical mitral valve implantation.

**Fig. 1:**
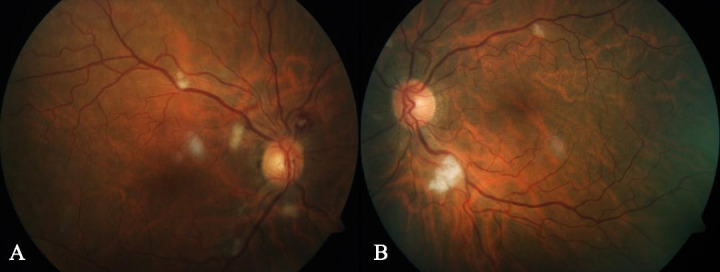
Fundus examination showing bilateral cotton-wool exudates and a Roth’s spot in his right eye

Visual acuity was 1 in both eyes using Snellen’s visual acuity chart. Anterior segment slit lamp examination showed no pathological signs and intraocular pressure was not altered. Fundus examination revealed bilateral cotton-wool exudates and a white-centred retinal haemorrhage compatible with a Roth’s spot in his right eye, suggesting retinal ischaemia (**Fig. 1**), not present in preoperative ophthalmological examination. 

**Fig. 2:**
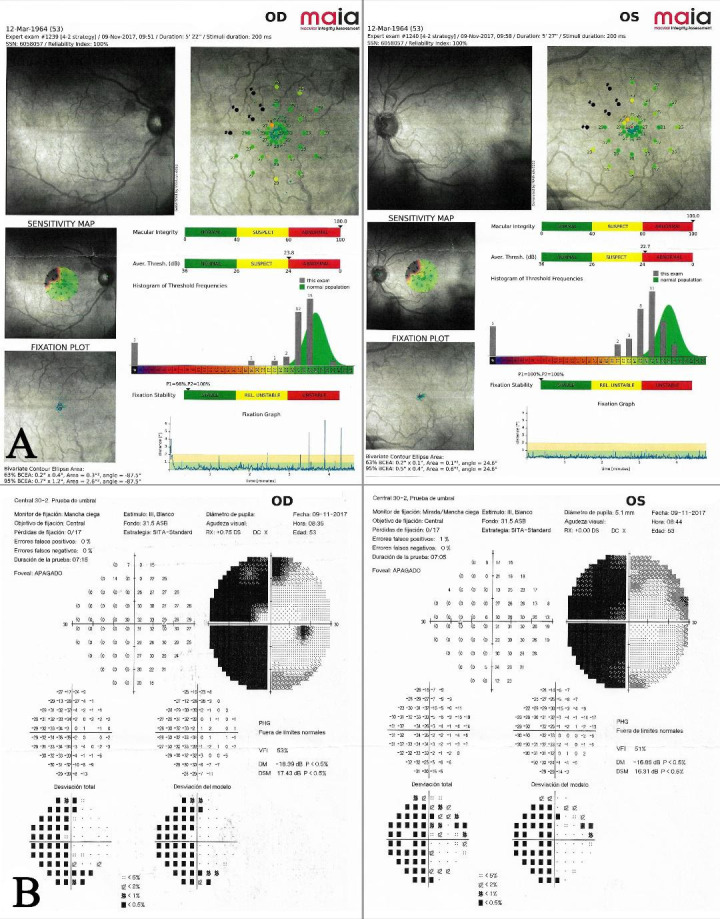
**A.** Visual field showing left homonymous hemianopia. **B.** Macular Integrity Assessment showing altered sensitivity map in right’s eye temporal area and in left eye’s nasal area

However, these lesions did not explain the patient’s clinical symptoms so a visual field campimetry and microperimetric examination using Macular Integrity Assessment were carried out. Left homonymous hemianopia was confirmed and microperimetric examination revealed altered values in the right eye’s temporal area and in the left eye’s nasal area, which were consistent with the visual field campimetry findings (**Fig. 2**). Neuroimaging was then requested and a right posterior cerebral artery occlusion was reported. 

As visual acuity was not affected and no macular oedema was present in OCT imaging, we decided to have a conservative attitude. The case was referred to the Neurology Department as ophthalmological findings were not responsible for the patient’s symptoms. They also decided to have a conservative attitude as the event had occurred over a week before.

**Fig. 3:**
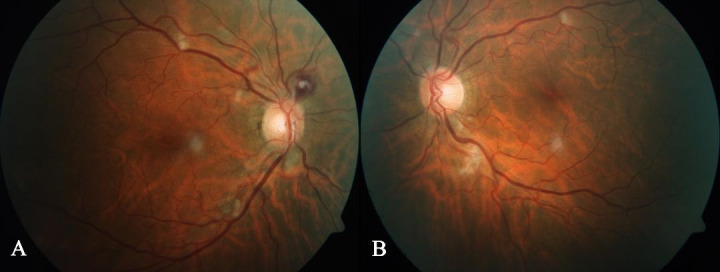
Fundus examination after second-week follow-up showing spontaneous remission of cotton-wool exudates, especially in left eye

Fundus lesions started to spontaneously remit, especially in his left eye, after the second week of follow-up (**Fig. 3**), although homonymous hemianopia persisted.

## Discussion

Cardiac surgery, especially when using extracorporeal circulation, has been linked to a variety of ocular post-operative manifestations. Optic nerve head blood flow has been proven to significantly decrease during these surgeries [**1**] and cases of ischemic optic neuropathy have been reported as a result of hypoperfusion [**2**]. Retinal artery occlusion risk has also been proven to be higher in open-heart surgeries compared to those non open-heart surgeries [**3**].

Other ophthalmological post-operative findings include diffuse chorioretinopathy [**4**], simultaneous bilateral retinal detachment after coronary artery bypass graft [**5**], retinal microembolisms demonstrated by fluorescein angiography [**6**], flame-shaped haemorrhages and soft exudates [**7**].

Impaired cerebral blood flow during cardiac surgery has also been described [**8**].

## Results

A combination of vascular complications linked to cardiac surgery were presented in this case report, both of which affected the visual system but originated in different tissues: retinal ischaemia and posterior cerebral artery occlusion.

Correlation between optic tract disease, visual field alterations and microperimetry changes were shown, highlighting the importance of microperimetry in order to determine the extent of macular involvement not clearly detailed in the visual field examination. This could have important prognostic implications as central values were altered in Macular Integrity Assessment tests, visual therapy techniques could be recommended in order to develop extrafoveal fixation and improve quality of life in these patients.

Carrying out a systematic ophthalmological examination or a vision capacity questionnaire in patients undergoing these surgeries is of great importance as waiting for the patient to complain about visual symptoms may sometimes be too late to act. 

## Conclusion 

Complications described highlighted the necessity of a higher control of hemodynamic parameters during cardiac surgery, especially when using extracorporeal circulation, in order to minimize peripheral tissues from suffering.

**Conflict of interest**

Authors declare no conflict of interest.

**Funding**

There are no funders to report for this submission.

**Informed consent**

Consent was gathered from the patient in order to obtain and publish these images.
